# *Helicobacter pylori* and oral–gut microbiome: clinical implications

**DOI:** 10.1007/s15010-023-02115-7

**Published:** 2023-11-02

**Authors:** Maged T. Elghannam, Moataz H. Hassanien, Yosry A. Ameen, Emad A. Turky, Gamal M. ELattar, Ahmed A. ELRay, Mohammed D. ELTalkawy

**Affiliations:** Hepatogastroenterology Department, Theodor Bilharz Research Institute, Giza, Egypt

**Keywords:** *H. pylori* infection, Oral and gut microbiota, Peptic ulcer disease, Gastric carcinoma, Clinical implications

## Abstract

More than half of the world’s population are colonized with H. pylori; however, the prevalence varies geographically with the highest incidence in Africa. *H. pylori* is probably a commensal organism that has been associated with the development of gastritis, ulcers, and gastric cancer. *H. pylori* alone is most probably not enough for the development of gastric carcinoma, but evidence for its association with the disease is high and has, therefore, been classified by the International Agency for Research on Cancer as a Class 1 carcinogen. Bacteroidetes and Fusobacteria positively coexisted during *H. pylori* infection along the oral–gut axis. The eradication therapy required to treat *H. pylori* infection can also have detrimental consequences for the gut microbiota, leading to a decreased alpha diversity. Therefore, therapy regimens integrated with probiotics may abolish the negative effects of antibiotic therapy on the gut microbiota. These eradication therapies combined with probiotics have also higher rates of eradication, when compared to standard treatments, and are associated with reduced side effects, improving the patient’s compliance. The eradication therapy not only affects gut microbiome but also affects the oral microbiome with robust predominance of harmful bacteria. However, there have been reports of a protective role of *H. pylori* in Barrett’s esophagus, esophageal adenocarcinoma, eosinophilic esophagitis, IBD, asthma, and even multiple sclerosis. Therefore, eradication therapy should be carefully considered, and test to treat policy should be tailored to specific communities especially in highly endemic areas. Supplementation of probiotics, prebiotics, herbals, and microbial metabolites to reduce the negative effects of eradication therapy should be considered. After failure of many eradication attempts, the benefits of *H. pylori* eradication should be carefully balanced against the risk of adverse effects especially in the elderly, persons with frailty, and intolerance to antibiotics.

## Background

*H. pylori* is a commensal organism associated with the development of gastritis, ulcers, and gastric cancer. The organism as well as eradication remedies can modulate gut microbiota in humans. Other non-*H. pylori* microbial species may colonize the same milieu, but *H. pylori* are regarded as a human pathogen [1]. Interactions between *H. pylori* and other members of the microbiome, the host, and the environment influence the clinical consequence and may lead to either disease or possible protective effects. Considering the beneficial effects on the host by regulating gastrointestinal microbiota, eradication of *H. pylori* can produce various adverse effects and alter the gastrointestinal microbiota. Gastrointestinal microbiota are defined as the entire community of microorganisms dwelling in the gastrointestinal tract, and it is dominated substantially by bacteria [2]. Gut microbiota composition varies between ethnic groups due to the different dietary, hygienic, and genetic factors in addition to the use of antibiotics. Its homeostasis plays a critical role in maintaining host health. Dysbiosis of the gut microbiome may produce multiple diseases and bacterial infections in addition to compromising human alimentation [3]. This review aims to discuss the relationships between *H. pylori* alone and in combination with oral and gut microbiota in the development of GI disease.

### General characteristics of *H. pylori*

*H. pylori* colonization affects more than half of the population worldwide [4] with the highest incidence in Africa (79.1%) [5]. Despite this high prevalence, the majority of the infected population are asymptomatic. Acquisition of *H. pylori *occurs in early childhood (30%–50%), while during early adolescence, it reaches over 90% in developing countries [6]. The consequence of infection varies either from no clinical symptoms or continuing throughout his life with superficial chronic gastritis [7, 8] or developing peptic ulcers, 25% even experience ulcer complications, and 1% will advance to gastric cancers (GC) [9].

The main transmission route of *H. pylori* is not known. However, the intrafamilial transmission of the pathogen is the most significant route. This may be facilitated by close personal contacts, the unified socioeconomic status of the family members, and the genetic predisposition to *H. pylori* persistence [10, 11].

Children < 5 years of age have high infection rates, after which infection declines at school age when less time is spent at home. In Egypt, 33% of children < 6 years are infected [12]. The disease development is influenced by several factors such as host genetics, environmental factors related to diets, lifestyle habits, and pathogens [13].

Once entering the stomach, *H. pylori* produces urease to convert urea to ammonia which neutralizes hydrochloric acid, then after, uses mobile flagella to spread over the surface of the gastric epithelium forming microbial biofilms. Gastric persistence is determined mainly by bacterial adhesion [14]. The adhesion of *H. pylori* is facilitated by the gastric epithelium α1,2-Fucosylated glycans [15]. Both virulence factors; cytotoxic-associated gene, (CagA) and vacuolating cytotoxin A (vacA), have direct damaging impact on the gastric mucosal epithelium [16, 17]. The initial host Th1 cell immune response intended to eradicate the microorganisms is opposed by *H. pylori* vacA immunosuppressive effect [18]. A Th2 cellular pathway facilitates *H. pylori* colonization in infancy and leads to the development of immune tolerance resulting in a symbiotic relationship between the microbe and the host [19]. *H. pylori* influence host immune responses and the microbiota of both the stomach and distal organs [20].

The persistence of *H. pylori* in the stomach is associated with the development of gastroduodenal diseases, such as chronic gastritis, peptic ulcer disease (PUD), gastric adenocarcinoma, and gastric MALT lymphoma, and colorectal carcinoma [21–23]. A significant relationship between *H. pylori* detection and pancreatic cancer has also been reported [23–25]. *H. Pylori* had been implicated in the pathogenesis of extra-gastric diseases such as ischemic brain injury [26], Alzheimer’s disease [27, 28], Parkinson’s disease [29], atherosclerotic vascular lesions, a higher risk of coronary heart disease [30–33], hypertension [34], endothelial dysfunction [34], vitamin B12 and folic acid malabsorption [35, 36]. Psoriasis [37, 38], lichen ruber planus, scabies, rosacea, Sweet’s syndrome, Behcet’s disease, and Schönlein-Henoch purpura [39] The mechanism of this extra gastric affection is not confirmed; however, *H. pylori* generates local inflammation in the stomach and can spread systemically by the release of cytokines establishing low-grade and chronic inflammation throughout the body [40].

In contrast to the harmful effects, *H. pylori* found to have a protective effect against many pathological conditions such as IBD [41, 42], asthma [43–47], esophageal adenocarcinoma [48], eosinophilic esophagitis [49], and GERD and Barrett’s esophagus [50, 51].

### *Helicobacter pylori *and oral microbiota

*H. pylori* detected in both the mouth and gut. The oral–gut axis microbiota has a dominant effect in *H. pylori’s* colonization, infection, and pathogenicity [52]. The number of *H. pylori* in the mouth is lower than in the stomach. It constitutes 42%–97% of the total gastric bacterial community [53]. The oral and gastric milieus are affected by saliva and digested food. The oral microbiome is the dominant source of gastric microbes, so, it is accused for the infection and transmission of *H. pylori* [54, 55].

The interplay between *H. pylori* and oral microbiome may take one or further of three main forms: co-aggregation, symbiotic biofilm formation, and endosymbiosis [56]. Fusobacterium nucleatum and *Porphyromonas gingivalis* are crucial bacteria in periodontal infection. The aggregation with *H. pylori* promotes oral to gastric colonization by oral bacteria [57]. Biofilms are surface bacterial communities embedded within an extracellular matrix. They contribute to an infection becoming chronic or recurrent, promote inflammation, and can make bacterial colonies resistant to antibiotics and the immune system [58].

The major cariogenic bacterium, Streptococcus mutans, forms a symbiotic biofilm with *H. pylori *prolonged its survival in the unsuitable atmosphere of the mouth [59]. *H. pylori *can anchor on the surface and/or enter *C. albicans* to form a mixed biofilm in the oral cavity and vagina [60, 61].

The interaction between and *H. pylori* members of the oral microbial community yields different results according to oral or gastrointestinal complaints in *H. pylori*-positive people. *P. gingivalis *has been established as a pathogenic agent of periodontitis and positively associated with *H. pylori*, indicating that *H. pylori* infection may promote periodontal disease [62]. The inter-transmission between oral and gut microorganisms can affect the ecosystem in both territories and hence regulate the pathogenesis of different diseases [63].

### Gastric bacterial microbiome profile

**Fig. 1:**
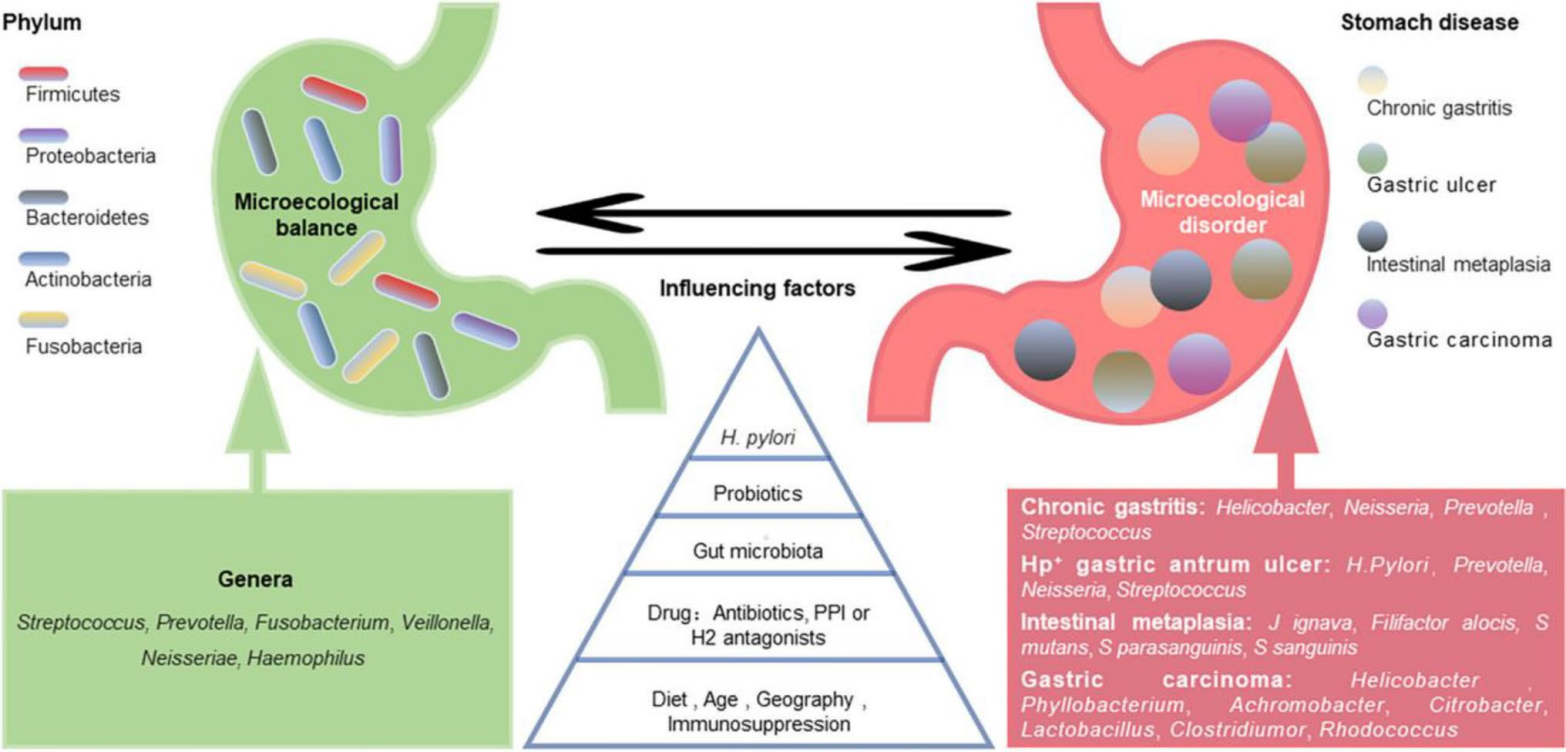
Gastric micro ecological imbalance and gastric diseases. Despite the differences among individuals, there are five dominant bacterial phyla in the healthy stomach, and their common dominant bacterial genera are summarized (green). The gastric microbiota is dynamically balanced and affected by many factors, such as *Helicobacter pylori* infection, probiotics, gut microbiota, drugs, diet, and age. Although the causal relationship between them is unclear, gastric micro ecological imbalances are associated with various gastric diseases (red), and some microorganism-related disorders are listed. With permission from Zhang L et al. published in Front. Microbiol 2023 “Gastric microbiota dysbiosis and *Helicobacter pylori* infection”

The gastric core microbiome is mainly formed from five major phyla, including Firmicutes, Bacteroidetes, Actinobacteria, Fusobacteria, and Proteobacteria [64, 65]. Numerous oral bacteria such as Neisseria, Veillonella, Fusobacterium, Streptococcus, and Hemophilus, are enriched in the lower digestive tract and can be significantly found in gastric biopsy too [66]. A healthy gut microbiome is characterized by high gut microbial diversity [67]. The co-occurrence interactions were stronger in intestinal metaplasia (IM) than superficial gastritis (SG) [68] and among genera in IM which then decreased in intraepithelial neoplasia (IN) of gastric biopsies with gastritis progression suggesting that the bacteria tend to co-occur to form a specific micro ecology before the occurrence of neoplasia [69]. Several factors can affect the gastric microbiome such as diet, aging, geographic area of residence, and medications like PPI and antibiotics [70, 71]. A reduced number of Bacteroidetes and elevated numbers of Firmicutes and Proteobacteria were observed in patients with gastritis as compared with healthy individuals [72] (Fig. 1).

### Gut microbiota and *Helicobacter pylori* infection

**Fig. 2:**
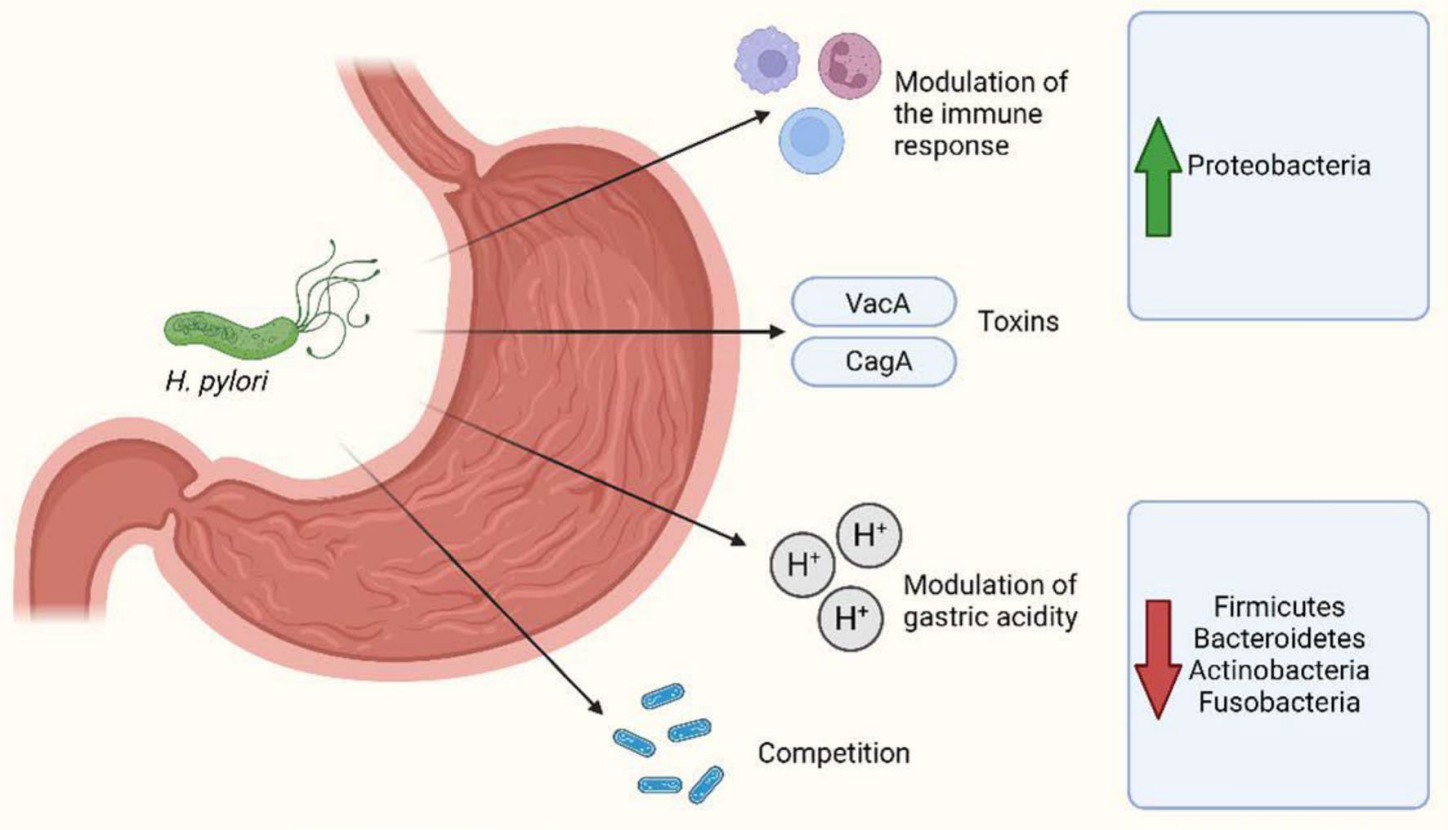
Main mechanisms mediating the relationship between *H. pylori* and gastric microbiota. Created with BioRender.com

*H. pylori* infection disturbs commensal bacterium equilibrium in the gastric mucosa in addition to the disturbance of microbial changes in the human gut [73–76]. *H. pylori* mainly influences the microbial composition and diversity in gastric mucosa rather than both gastric juice and stool [69] *H. pylori* infection results concerning bacterial diversity have been controversial as has been found with other specific groups of gut bacteria [77, 78]. *Lactobacillus* species abundance was higher in *H. pylori*-infected patients than in non-infected persons [79] , protecting the human gut from bacterial colonization through gut barrier preservation [80]. Iino et al. in 2018 [79] found that *H. pylori*-positive patients displayed reduced amounts of *L. acidophilus* and an increased proportion of *L. salivarius* in comparison with non-infected subjects due to the suppression of gastric acid secretion by *H. pylori* infection. He reported a higher abundance of *Lactobacillus* in *H. pylori*-positive patients with severe atrophic gastritis compared to infected patients with mild atrophic gastritis or without gastritis denoting affection of gastric microbiota according to symptom severity. On the other hand, gut bacteria might also influence the bacterial colonization of other gastrointestinal regions, including *H. pylori* in the stomach. Nitrospirae phylum can be seen only in *H. pylori*-negative personnel with minimal values in patients with duodenal ulcer and *H. pylori* infection as nitrite has a bactericidal effect against *H. pylori* [80, 81]. *H. pylori* infection alters the gut microbiota in asymptomatic patients by increasing Proteobacteria, *Clostridium*, *Firmicutes*, and *Prevotella* in a pediatric population [78] and members belonging to Succinivibrio, Coriobacteriaceae, Enterococcaceae, and Rikenellaceae in adults [75] compared to non-infected subjects. Gao et al. in 2018 [72] reported a disturbance of fecal microbiota, mainly the phyla Bacteroidetes, *Firmicutes*, and Proteobacteria in *H. pylori*-induced gastric diseases. The relationship between *H. pylori* and gastric microbiota could be mediated through multiple mechanisms, such as virulence factors, the modification of gastric acidity, host immune responses, and competition [82] (Fig. 2).

*H. pylori*-resistant strains showed a higher trend of diversity and evenness than the sensitive samples. The abundance of resistant strains decreased with increasing cohabitation of pathogenic bacteria. There is an increase in the α-diversity index among the MDR. The resistance status of *H.* *pylori* was correlated with the enriched diversity of the gastric microbiome composition, where the abundance of non-pylori pathogens increased, especially in triple-resistant strains [83].

### Gut microbiota and *Helicobacter pylori *eradication therapy

Antibiotic administration decreases bacterial diversity [84, 85]. Bacterial diversity was restored in the short and the long term after treatment conclusion [86–89]. Still, not all studies reported enhancement in bacterial diversity after treatment conclusion [90, 91]. Generally, gut microbiota composition is restored in most cases at 2 months post-treatment. Proteobacteria phylum is proposed to be partially responsible for the development of adverse effects during eradication therapy [92]. Probiotic supplementation and the antibiotic impose a beneficial gut microbiota profile after eradication therapy [93]. Niu et al. in 2021 [94] reported a success rate of *H. pylori* eradication 95.5% using the quadruple remedy. The majority of phyla in the two groups were the same and included Proteobacteria, Bacteroides, *Firmicutes*, Actinomycetes, and *Fusobacteria*. The microbial diversity in the failure group had a lowering fashion and the species abundance became extensively reduced compared with the success group. The presence of Rhodococcus, *Lactobacillus*, and *Sphingomonas* was associated with high rate of *H. pylori* eradication in the successful group. Veronococcus and Cilium were enriched in the mucosa of chronic atrophic gastritis cases compared to chronic superficial gastritis cases. In both study groups, *H. pylori* were negatively identified with other bacterial groups. They concluded that gastric microbiota is the corner stone in the effect of quadruple *H. pylori* eradication therapy. Tawfik et al. in 2023 [95] found oral microbiomes more diverse than the gut microbiomes. The eradication of *H. pylori* was associated with a significant reduction in the bacterial diversity along the orointestinal axis. *H. pylori* positive patients showed positive correlation between Proteobacteria and Fusobacteria. After eradication therapy, *Fusobacterium*, *Veillonella*, *Catenibacterium*, *Neisseria* and *Prevotella* enriched significantly. They stress the importance of eradication therapy on certain genera especially, in the oral microbiota.

### *Helicobacter pylori*-associated diseases

#### Gastritis and ulcer disease

Only 10% of the population develop clinical manifestations latterly in their lives when getting elderly [96]. Seventy percent of people who are established to have the bacterium are healthy bacterial carriers, and 5%–10% of those infected develop symptoms of gastritis or PUD [97–101]. Absence of *H. pylori*-gastritis had been reported [102, 103] , and indeed in severe cases and premalignant conditions, a low abundance of *H. pylori* had been reported [104]. *H. pylori*-negative gastritis was found to be 21% in the United States [105] and 27% of all cases of gastritis in Indonesia [103]. Araújo et al. in 2014 [106] reported that the discovery rate of *H. pylori* infection in cases diagnosed with PUD is the same as in the general population and 20–50% of PUD patients had idiopathic etiology. The high prevalence rate and low incidence of pathological diseases indicate that *H. pylori* are more likely to be an opportunistic or latent pathogen rather than a truly pathogenic bacterium. The development of PUD is multifactorial and depends on endogenous and exogenous factors, which means that the presence of *H. pylori* infection may be only one of many factors involved in the genesis of ulcerative disorders. The genotype of *H. pylori* is a determinant factor in producing ulcer disease. Cases with a verified diagnosis of PUD had vacA-positive and CagA-positive genotypes [107, 108]. Our group [109] reported a low prevalence of CagA (26.5%). Western type CagA is the fundamental kind (62.5%) while the East Asian type was not detected and others (37.5%) remain uncharacterized. Western-genotype CagA was found in 80% of patients with peptic ulcer disease and 40% of patients with gastritis. The primary genotype mixture in the studied Egyptian sufferers were*; vacAs2m2/iceA1, vacAs1m1/cagA*, mostly related to gastritis, and *vacAs1/cagA/icA*, mainly in PUD. The much less virulent *(s2, s2m2) H. pylori* genotypes were found in cases over the age of 43 years [110]. Lately, there has been a progressive increase in the idiopathic forms of PUD with a drop in the global frequencies of *H. pylori* infection. A further study demonstrated a significant correlation between the isolation of Streptococci and peptic ulcer disease [111]. Iijima and his associates [112] reported that 45.9% of cases of peptic ulcers of the stomach and 29.6% of those of the duodenum were idiopathic. *H. pylori*-positive ulcer had better convalescence rates, better course, more positive prognosis, less hospital stay, less 30 days readmission, and fewer recurrence rates [113, 114].

#### Gastric carcinoma

Gastric carcinoma (GC) develops in *H. pylori*-infected people 1.4–4.2 times more often than within the general population [115–117]. Even so, only 1–2% of cases develop GC in 50% or more of *H. pylori*-infected patients [118]. In spite of the superiority of *H. pylori* in Africa and India than in the West, the incidence of GC is less frequent than in the West [119], which is known as an epidemiologic paradox [120]. This decreasing trend of bacterial richness going from the normal tissue to peritumoral and tumoral tissues indicated that as the microenvironment of a tumor is altered, it becomes unsuitable for colonization with specific bacteria. The low microbial diversity of the upper digestive tract was associated with a low serum pepsinogen I/pepsinogen II ratio, which has also been associated with gastric carcinogenesis [121]. *H. pylori* virulence factors have not been reported to be essential for cancer development [1]. In an Egyptian study of the prevalence of *H. pylori* CagA among patients with gastric carcinoma, a total of 34 (56.67%) patients have been CagA + ve and 26 (43.33%) patients were CagA − ve, with no statistically significant difference regarding sex or age [122]. It is well known that the persistence of *H. pylori* infection is linked to the development of only non-cardiac carcinoma, while it had a protective effect against cancer development in the cardiac area and lower esophagus [48]. The decrease in *H. pylori* infections in Japan is believed to have contributed to a decline in gastric cancer cases [123]. However, the cause of an increase in gastric cancer in the young population in the USA (notably, young Hispanic men), where overall the incidence of *H. pylori* infection is also waning, is unexplained [124]. Thus, unknown factors likely unrelated to *H. pylori* infection may be contributing to a rise in gastric cancer in specific populations.

Recently, it is accepted that cancer pathogenesis is precipitated by confounding factors such as high-salt diets and other carcinogenic substances that promote the carcinogenic pathway in addition to bacterial agents [125]. The multistep processes involved in the development of GC are initiated by the transition of the mucosa into chronic non-atrophic gastritis, which is primarily triggered by infection with *H. pylori*. This gastritis then progresses into atrophic gastritis and intestinal metaplasia, and then to dysplasia, and following Correa’s cascade, to adenocarcinoma [119].

**Fig. 3:**
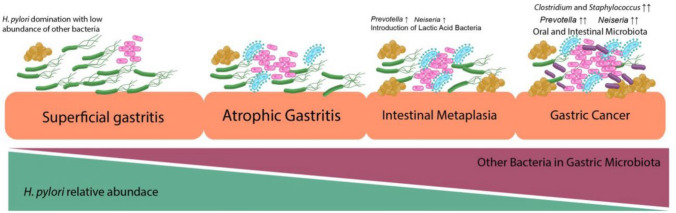
Association of *Helicobacter pylori* abundance with the different stages of gastric conditions. The presence of *H. pylori* was dominant in the superficial gastritis condition; thus, this domination reduced microbial diversity. In atrophic gastritis and intestinal metaplasia, the relative abundance of *H. pylori* began to decrease with the introduction of other bacteria, including the incremental of *Prevotella* sp. and *Neisseria* sp. In the gastric cancer condition, *H. pylori* started to deteriorate with a significantly increased amount other bacteria, including oral cavity microbiota, intestinal microbiota, and lactic acid bacteria. Published in Gut Pathogens (2022) 14:19 with permission

During the transition from gastritis to GC, a significant difference in the gastric microbial community was observed. There is an increase in the abundance of non-*H. pylori* proteobacteria [126] (Fig. 3).

An analysis of gastric microbial communities from different stages of gastric cancer development revealed the significance of Peptostreptococcus stomatis, *S. anginosus, Parvimonas micra*, Slackia exigua, and Dialister pneumosintes in the progression of gastric cancer, as they were found in the precancerous stage [68]. Gastric cancer prevalence varies among different regions of the stomach, with cancers arising in the corpus potentially caused by mechanisms distinct from the other regions [127]. Alternatively, physiological factors that vary along the length of the stomach and pylorus such as differences in oxygen concentration, pH, mucus, and nutrient availability could play a part in determining regional cancer susceptibility [128].

Ralser and his colleagues in 2023 identified a unique *H. pylori*-driven immune alteration signature characterized by a reduction in regulatory T cells in addition to *H. pylori* induction of pro-carcinogenic STAT3 signaling and a loss of goblet cells in colonic epithelium, changes that have been shown to contribute; in combination with pro-inflammatory and mucus degrading microbial signatures, to tumor development in the intestinal and colonic epithelium [129].

Metabolites and their interactions with microbiota may be involved in *Helicobacter pylori*-associated gastric lesion development. Negative correlations between Helicobacter and glycerophospholipids, glycosylceramide, and triacylglycerol, which were altered by eradication. The characteristic negative correlations between glycosylceramides and Fusobacterium, Streptococcus, and Gemella in *H. pylori*-positive baseline biopsy specimens were further noticed in active gastritis and intestinal metaplasia. This helps discriminate high-risk subjects for progression from mild lesions to advanced precancerous lesions in short-term and long-term follow-up [130].

Antibiotic treatment in *H. pylori*-infected patients can reduce GC progression even if *H. pylori* is not eradicated, suggesting that suppression of other bacteria may serve a protective function [131]. Niikura and his colleagues in 2023 [132] identified potential pathogens; abnormally colonized gastric bacteria, particularly Fusobacterium and *Neisseria *spp., play an additional fundamental role in the later stages of gastric carcinogenesis. Testing for Fusobacterium and *Neisseria spp*. in gastric mucosal samples as a surrogate for gastric dysbiosis could be a next-generation approach for screening high-risk patients for GC. In addition, eradication of these oncogenic bacteria and/or inhibition of carcinogenic bacteria-derived molecules may be a future strategy for preventing GC development, particularly in patients with severe atrophic gastritis and intestinal metaplasia.

#### Clinical implications

*H. pylori* infection is frequent in developing countries and represents an annoying health problem. Eradication remedies had been recommended by all societies for fear of malignancy. Eradication remedies are complicated by the requirement for several agents such as the use of 2 antibiotics and a PPI. These strategies can be complicated by antibiotic resistance, high cost on the national level, PPI-related complications, and not the least microbiota dysbiosis. Despite the high frequency of *H. pylori* infection, there is a low prevalence of gastric malignancy. *H. pylori* alone is most probably not enough for the development of GC. Considering the protective role of *H. pylori* against numerous conditions such as IBD, asthma, multiple sclerosis, Barrett’s esophagus, esophageal adenocarcinoma, and eosinophilic esophagitis, *H. pylori* is now considered one of the bacteria in the healthy microbiome for the majority of the human population. Therefore, not every case should be treated for eradication. A personalized approach according to the *H. pylori* indigenous region, the presence of gastrointestinal malignancies among relatives, or the impossibility of banning non-modifiable threat factors is needed. It should include relatives with high familial risk or living in high-risk areas/populations where eradication effectively reduces the threat of gastric carcinoma as in South East Asia and cases with cancer who are on therapy with immune checkpoint inhibitors or vaccine-grounded immunotherapy [133].

The supplementation of probiotics, prebiotics, and microbial metabolites to reduce the negative effects of eradication should be considered.

Probiotics reduce *H. pylori*-induced gastric pathology in mice, with reduced inflammatory infiltration and precancerous lesion incidence [134], enhance *H. pylori* eradication rates, and reduce side effects in humans [135].

Autoprobiotics refer to indigenous bifidobacteria, lactobacilli, or enterococci isolated from a specific individual, intended to restore microbiota and improve health. The advantages of autoprobiotics include its safety, high survival rate, its unique individual composition and, extended duration in the gut [136]. Both the quadruple therapy group and the *H. pylori*-negative subjects after probiotic-supplemented eradication treatment had nearly the same microbial diversity [137]. The most effective types belong to the Firmicutes (*Enterococcus* and *Lactobacillus*) and Actinobacteria (*Bifidobacterium* genus) phyla and Saccharomyces boulardii [138, 139]. However, Yang and his colleagues in 2021 [140] reported failure to improve the eradication rate of *H. pylori* after supplementation with lactobacillus, but it helped build up a beneficial microbial profile and reduced the frequencies of abdominal distention and diarrhea.

The potential mechanisms of probiotic action against *H. pylori* include correction of the gut microbiota, immunological effects such as enhancement of humoral and cellular immunity, and reduction of oxidative stress, direct antagonistic effects against *H. pylori* such as colonization resistance and bacteriocin synthesis, and stimulation of local immunological protection such as strengthening of the mucous protective barrier and reduction of gastric mucosa inflammation [141]. As a double-edged sword, the use of probiotic-induced adverse effects include higher risk of systemic neonates infections throughout their life span [142], long-term gut dysbiosis [143], and risk to develop Parkinson’s disease mostly due to Desulfovibrio bacteria [144, 145]. The oral administration of multi-strain probiotics and paraprobiotics were more than single-strain probiotics, reducing the incidence of developing metabolic disorders [146].

Washing microflora transfer (WMT) is a modified FMT method that uses washed preparations. Ye et al. in 2020 [147] reported that WMT has an overall *H. pylori* eradication of 40.6%. No *H. pylori* transmission was recorded from healthy, asymptomatic donors to recipients by oral capsule-based FMT [148].

*H. pylori* living in both oral cavity and gut looks to be a commensal, occasionally pathogenic. It is not surprising for a case to have more than an eradication course. This is harmful to both oral and gut microbiota and may lead to different diseases. After multiple failed eradication attempts, the implicit benefits of *H. pylori* eradication should be weighed against the liability of adverse effects with repeated high-dose acid suppression and antibiotic exposure, particularly among individuals who are not at an identifiably advanced threat of complications from persistent *H. pylori* infection such as, GC or peptic ulcer disease. Similarly, a careful decision-making approach should be seriously considered, especially in the senior, those with frailty, and those with intolerance to antibiotics [149] (Best Practice Advice #9).

## References

[1] Sitkin S, Lazebnik L, Avalueva E, Kononova S, Vakhitov T (2022). Gastrointestinal microbiome and *Helicobacter pylori*: eradicate, leave it as it is, or take a personalized benefit–risk approach?. World J Gastroenterol.

[2] Ley RE, Peterson DA, Gordon JI (2006). Ecological and evolutionary forces shaping microbial diversity in the human intestine. Cell.

[3] Azad M, Sarker M, Li T, Yin J (2018). Probiotic species in the modulation of gut microbiota: an overview. Biomed Res Int.

[4] Malnick SD, Melzer E, Attali M, Duek G, Yahav J (2014). *Helicobacter pylori*: friend or foe?. World J Gastroenterol.

[5] Hooi JKY, Lai WY, Ng WK, Suen MMY, Underwood FE, Tanyingoh D (2017). Global prevalence of *Helicobacter pylori* infection: systematic review and meta-analysis. Gastroenterology.

[6] Go MF (2002). Review article: natural history and epidemiology of *Helicobacter pylori* infection. Aliment Pharmacol Ther.

[7] Hunt RH (1996). The role of *H. pylori* in pathogenesis: the spectrum of clinical outcomes. Scand J Gastroenterol.

[8] Rothenbacher D, Brenner H (2003). Burden of *Helicobacter pylori* and *H. pylori*-related diseases in developed countries: recent developments and future implications. Microbes Infect.

[9] Malaty HM (2007). Epidemiology of *Helicobacter pylori* infection. Best Prac Res Clin Gastroenterol.

[10] Kayali S, Manfredi M, Gaiani F, Bianchi L, Bizzarri B, Leandro G (2018). *Helicobacter pylori*, transmission routes and recurrence of infection: state of the art. Acta Biomed.

[11] Sgambato D, Visciola G, Ferrante E, Miranda A, Romano L, Tuccillo C (2018). Prevalence of *Helicobacter pylori* infection in sexual partners of *H. pylori-*infected subjects: role of gastroesophageal reflux. United Eur Gastroenterol J.

[12] Frenck RW, Fathy HM, Sherif M, Mohran Z, El Mohammedy H, Francis W (2006). Sensitivity and specificity of various tests for the diagnosis of *Helicobacter pylori* in Egyptian children. Pediatrics.

[13] Waskito L, Rezkitha Y, Vilaichone R, Sugihartono T, Mustika S, Wibawa D (2022). The role of non-*Helicobacter pylori* bacteria in the pathogenesis of gastroduodenal diseases. Gut Pathogens.

[14] Kao CY, Sheu BS, Wu JJ (2016). *Helicobacter pylori* infection: an overview of bacterial virulence factors and pathogenesis. Biomed J.

[15] Goto Y, Uematsu S, Kiyono H (2016). Epithelial glycosylation in gut homeostasis and inflammation. Nat Immunol.

[16] Chang WL, Yeh YC, Sheu BS (2018). The impacts of *H. pylori* virulence factors on the development of gastroduodenal diseases. J Biomed Sci.

[17] Ricci V, Giannouli M, Romano M, Zarrilli R (2014). *Helicobacter pylori *gamma-glutamyl transpeptidase and its pathogenic role. World J Gastroenterol.

[18] Altobelli A, Bauer M, Velez K, Cover TL, Müller A (2019). *Helicobacter pylori* VacA targets myeloid cells in the gastric lamina propria to promote peripherally induced regulatory T-cell differentiation and persistent infection. MBio.

[19] D'Elios MM, Codolo G, Amedei A, Mazzi P, Berton G, Zanotti G (2009). *Helicobacter pylori*, asthma, and allergy. FEMS Immunol Med Microbiol.

[20] Kienesberger S, Cox LM, Livanos A, Zhang XS, Chung J, Perez-Perez GI (2016). Gastric *Helicobacter pylori* infection affects local and distant microbial populations and host responses. Cell Rep.

[21] Sokic-Milutinovic A, Alempijevic T, Milosavljevic T (2015). Role of *Helicobacter pylori* infection in gastric carcinogenesis: current knowledge and future directions. World J Gastroenterol.

[22] Huang JQ, Sridhar S, Hunt RH (2002). Role of *Helicobacter pylori* infection and non-steroidal anti-inflammatory drugs in peptic ulcer disease: a meta-analysis. Lancet.

[23] Papamichael K, Konstantopoulos P, Mantzaris GJ (2014). *Helicobacter pylori* infection and inflammatory bowel disease: is there a link?. World J Gastroenterol.

[24] Xiao M, Wang Y, Gao Y (2013). Association between *Helicobacter pylori* infection and pancreatic cancer development: a meta-analysis. PLoS ONE.

[25] Ertz-Archambault N, Keim P, Von Hoff D (2017). Microbiome and pancreatic cancer: a comprehensive topic review of the literature. World J Gastroenterol.

[26] Wang ZW, Li Y, Huang LY, Guan QK, Xu DW, Zhou WK (2012). *Helicobacter pylori* infection contributes to high risk of ischemic stroke: evidence from a meta-analysis. J Neurol.

[27] Kountouras J, Boziki M, Zavos C, Gavalas E, Giartza-Taxidou E, Venizelos I (2012). A potential impact of chronic *Helicobacter pylori* infection on Alzheimer's disease pathobiology and course. Neurobiol Aging.

[28] Franceschi F, Covino M, Roubaud Baudron C (2019). Review: *Helicobacter pylori* and extra-gastric diseases. Helicobacter.

[29] Huang HK, Wang JH, Lei WY, Chen CL, Chang CY, Liou LS (2018). *Helicobacter pylori* infection is associated with an increased risk of Parkinson's disease: a population-based retrospective cohort study. Parkinsonism Relat Disord.

[30] Izadi M, Fazel M, Sharubandi SH, Saadat SH, Farahani MM, Nasseri MH (2012). Helicobacter species in the atherosclerotic plaques of patients with coronary artery disease. Cardiovasc Pathol.

[31] Park MJ, Choi SH, Kim D, Kang SJ, Chung SJ, Choi SY (2011). Association between *Helicobacter pylori* seropositivity and the coronary artery calcium score in a screening population. Gut Liver.

[32] Jukic A, Bozic D, Kardum D, Becic T, Luksic B, Vrsalovic M (2017). *Helicobacter pylori* infection and severity of coronary atherosclerosis in patients with chronic coronary artery disease. Ther Clin Risk Manag.

[33] Sharma V, Aggarwal A (2015). *Helicobacter pylori*: does it add to the risk of coronary artery disease?. World J Cardiol.

[34] Vijayvergiya R, Vadivelu R (2015). Role of *Helicobacter pylori* infection in the pathogenesis of atherosclerosis. World J Cardiol.

[35] Santarelli L, Gabrielli M, Cremonini F, Santoliquido A, Candelli M, Nista EC (2004). Atrophic gastritis as a cause of hyperhomocysteinaemia. Aliment Pharmacol Ther.

[36] Cárdenas VM, Boller F, Román GC (2019). *Helicobacter pylori*, vascular risk factors and cognition in US older adults. Brain Sci.

[37] Yu M, Zhang R, Ni P, Chen S, Duan G (2019). *Helicobacter pylori* infection and psoriasis: a systematic review and meta-analysis. Medicine (Kaunas).

[38] Onsun N, Arda Ulusal H, Su O, Beycan I, Biyik Ozkaya D, Senocak M (2012). Impact of *Helicobacter pylori* infection on the severity of psoriasis and response to treatment. Eur J Dermatol.

[39] Yorulmaz A, Kulcu SC (2015). *Helicobacter pylori* and inflammatory skin diseases. World J Dermatol.

[40] De Brito BB, Da Silva FAF, Soares AS, Pereira VA, Cordeiro Santos ML, Sampaio MM (2019). Pathogenesis and clinical management of *Helicobacter pylori* gastric infection. World J Gastroenterol.

[41] Kyburz A, Müller A (2017). *Helicobacter pylori* and extragastric diseases. Curr Top Microbiol Immunol.

[42] Wu XW, Ji HZ, Yang MF, Wu L, Wang FY (2015). *Helicobacter pylori* infection and inflammatory bowel disease in Asians: a meta-analysis. World J Gastroenterol.

[43] Chen Y, Blaser MJ (2008). *Helicobacter pylori* colonization is inversely associated with childhood asthma. J Infect Dis.

[44] Miftahussurur M, Nusi IA, Graham DY, Yamaoka Y (2017). Helicobacter, hygiene, atopy, and asthma. Front Microbiol.

[45] Tsigalou C, Konstantinidis TG, Cassimos D, Karvelas A, Grapsa A, Tsalkidis A (2019). Inverse association between *Helicobacter pylori* infection and childhood asthma in Greece: a case-control study. Germs.

[46] Elias N, Nasrallah E, Khoury C, Mansour B, Abu Zuher L, Asato V (2020). Associations of *Helicobacter pylori* seropositivity and gastric inflammation with pediatric asthma. Pediatr Pulmonol.

[47] Ierardi E, Losurdo G, Giorgio F, Di Leo A (2020). Might *Helicobacter pylori* play a role in allergic or cross-reaction-related disorders?. Expert Rev Gastroenterol Hepatol.

[48] Hansen S, Melby KK, Aase S, Jellum E, Vollset SE (1999). *Helicobacter pylori* infection and risk of cardia cancer and non-cardia gastric cancer. A nested case-control study. Scand J Gastroenterol.

[49] Shah S, Tepler A, Peek R, Colombel J, Hirano I, Narula N (2019). Association between *Helicobacter pylori* exposure and decreased odds of eosinophilic esophagitis-a systematic review and meta-analysis. Clin Gastroenterol Hepatol.

[50] Rubenstein J, Inadomi J, Scheiman J, Schoenfeld P, Appelman H, Zhang M (2014). Association between *Helicobacter pylori *and Barrett's esophagus, erosive esophagitis, and gastroesophageal reflux symptoms. Clin Gastroenterol Hepatol.

[51] Bor S, Kitapcioglu G, Kasap E (2017). Prevalence of gastroesophageal reflux disease in a country with a high occurrence of *Helicobacter pylori*. World J Gastroenterol.

[52] Chen X, Wang N, Wang J, Liao B, Cheng L, Ren B (2022). The interactions between oral gut axis microbiota and *Helicobacter pylori*. Front Cell Infect Microbiol.

[53] Schulz C, Schütte K, Koch N, Vilchez-Vargas R, Wos-Oxley ML, Oxley APA (2018). The active bacterial assemblages of the upper GI tract in individuals with and without helicobacter infection. Gut.

[54] Freitas D, Le Feunteun S, Panouille M, Souchon I (2018). The important role of salivary a-amylase in the gastric digestion of wheat bread starch. Food Funct.

[55] Wu ZF, Zou K, Xiang CJ, Jin ZJ, Ding HH, Xu S (2021). *Helicobacter pylori* infection is associated with the co-occurrence of bacteria in the oral cavity and the gastric mucosa. Helicobacter.

[56] Chen X, Zhou X, Liao B, Zhou Y, Cheng L, Ren B (2021). The cross-kingdom interaction between *Helicobacter pylori* and candida albicans. PloS Pathog.

[57] Park J, Shokeen B, Haake SK, Lux R (2016). Characterization of fusobacterium nucleatum ATCC 23726 adhesins involved in strain-specific attachment to porphyromonas gingivalis. Int J Oral Sci.

[58] Hathroubi S, Servetas SL, Windham I, Merrell DS, Ottemann KM (2018). *Helicobacter pylori* biofilm formation and its potential role in pathogenesis. Microbiol Mol Biol Rev.

[59] Nomura R, Kadota T, Ogaya Y, Matayoshi S, Iwashita N, Okawa R (2020). Contribution of streptococcus mutans to *Helicobacter pylori* colonization in the oral cavity and gastric tissue. Sci Rep.

[60] Palencia SL, Garcıa A, Palencia M (2022). Multiple surface interaction mechanisms direct the anchoring, co-aggregation and formation of dual-species biofilm between candida albicans, and *Helicobacter pylori*. J Adv Res.

[61] Saniee P, Siavoshi F, Nikbakht Broujeni G, Khormali M, Sarrafnejad A, Malekzadeh R (2013). Localization of *H. pylori* within the vacuole of candida yeast by direct immunofluorescence technique. Arch Iranian Med.

[62] Miller DP, Scott DA (2021). Inherently and conditionally essential protein catabolism genes of *p. gingivalis*. Trends Microbiol.

[63] Park SY, Hwang BO, Lim M, Ok SH, Lee SK, Chun KS (2021). Oral-gut microbiome axis in gastrointestinal disease and cancer. Cancers (Basel).

[64] Monstein HJ, Tiveljung A, Kraft CH, Borch K, Jonasson J (2000). Profiling of bacterial flora in gastric biopsies from patients with *Helicobacter pylori*-associated gastritis and histologically normal control individuals by temperature gradient gel electrophoresis and 16S rDNA sequence analysis. J Med Microbiol.

[65] Bik EM, Eckburg PB, Gill SR, Nelson KE, Purdom EA, Francois F (2006). Molecular analysis of the bacterial microbiota in the human stomach. Proc Natl Acad Sci USA.

[66] Zoetendal G, Raes J, van den Bogert B, Arumugam M, Booijink M, Troost J (2012). The human small intestinal microbiota is driven by rapid uptake and conversion of simple carbohydrates. ISME J.

[67] Le Chatelier E, Nielsen T, Qin J, Prifti E, Hildebrand F, Falony G (2013). Richness of human gut microbiome correlates with metabolic markers. Nature.

[68] Coker O, Dai W, Nie Z, Zhao J, Cao L, Nakatsu G (2018). Mucosal microbiome dysbiosis in gastric carcinogenesis. Gut.

[69] Liu D, Chen S, Gou Y, Yu W, Zhou H, Zhang R (2021). Gastrointestinal microbiota changes in patients with gastric precancerous lesions. Front Cell Microbiol.

[70] Nardone G, Compare D, Rocco A (2017). A microbiota-centric view of diseases of the upper gastrointestinal tract. Lancet Gastroenterol Hepatol.

[71] Hojo M, Asahara T, Nagahara A, Takeda T, Matsumoto K, Ueyama H (2018). Gut microbiota composition before and after use of proton pump inhibitors. Dig Dis Sci.

[72] Gao J, Zhang Y, Gerhard M, Mejias-Luque R, Zhang L, Vieth M (2018). Association between gut microbiota and *Helicobacter pylori*-related gastric lesions in a high-risk population of gastric cancer. Front Cell Infect Microbiol.

[73] Maldonado-Contreras A, Goldfarb KC, Godoy-Vitorino F, Karaoz U, Contreras M, Blaser MJ (2011). Structure of the human gastric bacterial community in relation to *Helicobacter pylori* status. ISME J.

[74] Lopetuso LR, Napoli M, Rizzatti G, Scaldaferri F, Franceschi F, Gasbarrini A (2018). Considering gut microbiota disturbance in the management of *Helicobacter Pylori* infection. Expert Rev Gastroenterol Hepatol.

[75] Dash NR, Khoder G, Nada AM, Al Bataineh MT (2019). Exploring the impact of *Helicobacter pylori* on gut microbiome composition. PLoS ONE.

[76] Frost F, Kacprowski T, Rühlemann M, Bang C, Franke A, Zimmermann K (2019). *Helicobacter pylori* infection associates with fecal microbiota composition and diversity. Sci Rep.

[77] He C, Peng C, Wang H, Ouyang Y, Zhu Z, Shu X (2019). The eradication of *Helicobacter pylori* restores rather than disturbs the gastrointestinal microbiota in asymptomatic young adults. Helicobacter.

[78] Benavides-Ward A, Vasquez-Achaya F, Silva-Caso W, Aguilar-Luis MA, Mazulis F, Urteaga N (2018). *Helicobacter pylori* and its relationship with variations of gut microbiota in asymptomatic children between 6 and 12 years. BMC Res Notes.

[79] Iino C, Shimoyama T, Chinda D, Arai T, Chiba D, Nakaji S (2018). Infection of *Helicobacter pylori* and atrophic gastritis influence lactobacillus in gut microbiota in a Japanese population. Front Immunol.

[80] Xiao S, Zhao L (2014). Gut microbiota-based translational biomarkers to prevent metabolic syndrome via nutritional modulation. FEMS Microbiol Ecol.

[81] Chen L, Xu W, Lee A, He J, Huang B, Zheng W (2018). The impact of Helicobacter pylori infection, eradication therapy and probiotic supplementation on gut microenvironment homeostasis: an open-label, randomized clinical trial. EBio Med.

[82] Fiorani M, Tohumcu E, Del Vecchio LE, Porcari S, Cammarota G, Gasbarrini A, Ianiro G (2023). The influence of *Helicobacter pylori* on human gastric and gut microbiota. Antibiotics.

[83] Dewayani A, Afrida Fauzia K, Alfaray RI, Waskito LA, Doohan D, Rejeki PS (2023). Gastric microbiome changes in relation with *Helicobacter pylori* resistance. PLoS ONE.

[84] Modi SR, Collins JJ, Relman DA (2014). Antibiotics and the gut microbiota. J Clin Invest.

[85] Reijnders D, Goossens GH, Hermes GDA, Neis EPJG, van der Beek CM, Most J (2016). Effects of gut microbiota manipulation by antibiotics on host metabolism in obese humans: a randomized double-blind placebo-controlled trial. Cell Metab.

[86] Jakobsson HE, Jernberg C, Andersson AF, Sjölund-Karlsson M, Jansson JK, Engstrand L (2010). Short-term antibiotic treatment has differing long-term impacts on the human throat and gut microbiome. PLoS ONE.

[87] Gotoda T, Takano C, Kusano C, Suzuki S, Ikehara H, Hayakawa S (2018). Gut microbiome can be restored without adverse events after *Helicobacter pylori* eradication therapy in teenagers. Helicobacter.

[88] Wu L, Wang Z, Sun G, Peng L, Lu Z, Yan B (2019). Effects of anti-*H. pylori* triple therapy and a probiotic complex on intestinal microbiota in duodenal ulcer. Sci Rep.

[89] Liou JM, Chen CC, Chang CM, Fang YJ, Bair MJ, Chen PY (2019). Long-term changes of gut microbiota, antibiotic resistance, and metabolic parameters after *Helicobacter pylori* eradication: a multicenter, open-label, randomized trial. Lancet Infect Dis.

[90] Martın-Núñez GM, Cornejo-Pareja I, Coin-Aragüez L, Roca-Rodrıguez MDM, Muñoz-Garach A, Clemente-Postigo M (2019). Pylori eradication with antibiotic treatment causes changes in glucose homeostasis related to modifications in the gut microbiota. PLoS ONE.

[91] Kakiuchi T, Mizoe A, Yamamoto K, Imamura I, Hashiguchi K, Kawakubo H (2020). Effect of probiotics during vonoprazan-containing triple therapy on gut microbiota in *Helicobacter pylori* infection: a randomized controlled Trial. Helicobacter.

[92] Hsu P-II, Pan C-YY, Kao JY, Tsay F-WW, Peng N-JJ, Kao S-SS (2018). *Helicobacter pylori* eradication with bismuth quadruple therapy leads to dysbiosis of gut microbiota with an increased relative abundance of proteobacteria and decreased relative abundances of bacteroidetes and actinobacteria. Helicobacter.

[93] Tang B, Tang L, Huang C, Tian C, Chen L, He Z (2021). The Effect of probiotics supplementation on gut microbiota after *Helicobacter pylori* eradication: a multicenter randomized controlled trial. Infect Dis Ther.

[94] Niu ZY, Li SZ, Shi YY, Xue Y (2021). Effect of gastric microbiota on quadruple *Helicobacter pylori* eradication therapy containing bismuth. World J Gastroenterol.

[95] Tawfik SA, Azab M, Ramadan M, Shabayek S, Abdellah A, Al Thagfan SS, Salah M (2023). The eradication of *Helicobacter pylori* was significantly associated with compositional patterns of orointestinal axis microbiota. Pathogens.

[96] Li J, Perez-Perez GI (2018). *Helicobacter pylori* the latent human pathogen or an ancestral commensal organism. Front Microbiol.

[97] Malfertheiner P, Chan FK, McColl KE (2009). Peptic ulcer disease. Lancet.

[98] Stein M, Ruggiero P, Rappuoli R, Bagnoli F (2013). *Helicobacter pylori* CagA: from pathogenic mechanisms to its use as an anti-cancer vaccine. Front Immunol.

[99] Calvet X, Ramírez Lázaro MJ, Lehours P, Mégraud F (2013). Diagnosis and epidemiology of *Helicobacter pylori* infection. Helicobacter.

[100] Walker MM, Talley NJ (2014). Review article: bacteria and pathogenesis of disease in the upper gastrointestinal tract–beyond the era of *Helicobacter pylori*. Aliment Pharmacol Ther.

[101] Tsimmerman YS (2019). Critical analysis of the *Helicobacter pylori*-infection leading role in the development of gastroduodenal diseases. Clin Pharmacol Ther.

[102] Shiota S, Thrift AP, Green L, Shah R, Verstovsek G, Rugge M, Graham DY, El-Serag HB (2017). Clinical manifestations of *Helicobacter pylori*-negative gastritis. Clin Gastroenterol Hepatol.

[103] Miftahussurur M, Waskito LA, Syam AF, Nusi IA, Wibawa ID, Rezkitha YA, Siregar G, Yulizal OK, Akil F, Uwan WB, Simanjuntak D (2019). Analysis of risks of gastric cancer by gastric mucosa among Indonesian ethnic groups. PLoS ONE.

[104] Pereira-Marques J, Ferreira RM, Pinto-Ribeiro I, Figueiredo C (2019). *Helicobacter pylori* infection, the gastric microbiome and gastric cancer. Adv Exp Med Biol.

[105] Nordenstedt H, Graham DY, Kramer JR, Rugge M, Verstovsek G, Fitzgerald S, Alsarraj A, Shaib Y, Velez ME, Abraham N, Anand B, Cole R, El-Serag HB (2013). *Helicobacter pylori*-negative gastritis: prevalence and risk factors. Am J Gastroenterol.

[106] Araújo MB, Borini P, Guimarães RC (2014). Etiopathogenesis of peptic ulcer: back to the past?. Arq Gastroenterol.

[107] Figura N, Guglielmetti P, Rossolini A, Barberi A, Cusi G, Musmanno RA, Russi M, Quaranta S (1989). Cytotoxin production by *Campylobacter pylori* strains isolated from patients with peptic ulcers and from patients with chronic gastritis only. J Clin Microbiol.

[108] Atherton JC, Cao P, Peek RM, Tummuru MK, Blaser MJ, Cover TL (1995). Mosaicism in vacuolating cytotoxin alleles of *Helicobacter pylori*. Association of specific vacA types with cytotoxin production and peptic ulceration. J Biol Chem.

[109] Diab M, Shemis M, Gamal D, El-Shenawy A, El-Ghannam M, El-Sherbini E (2018). *Helicobacter pylori* western cagA genotype in Egyptian patients with upper gastrointestinal disease. Egypt J Med Human Genetics.

[110] El-Shenawy A, Diab M, Shemis M, ElGhannam M, Salem D, Abdelnasser M (2017). Detection of *Helicobacter pylori* vacA, cagA and iceA1 virulence genes associated with gastric diseases in Egyptian patients. Egypt J Medi Human Genetics.

[111] Khosravi Y, Dieye Y, Poh BH, Ng CG, Loke MF, Goh KL (2014). Culturable bacterial microbiota of the stomach of *Helicobacter pylori* positive and negative gastric disease patients. Sci World J.

[112] Iijima K, Kanno T, Abe Y, Yagi M, Asonuma S, Ohyauchi M (2016). Preferential location of idiopathic peptic ulcers. Scand J Gastroenterol.

[113] Kanno T, Iijima K, Abe Y, Yagi M, Asonuma S, Ohyauchi M, Ito H, Koike T, Shimosegawa T (2016). *Helicobacter pylori*-negative and non-steroidal anti-inflammatory drugs-negative idiopathic peptic ulcers show refractoriness and high recurrence incidence: multicenter follow-up study of peptic ulcers in Japan. Dig Endosc.

[114] Rasane RK, Horn CB, Coleoglou Centeno AA, Fiore NB, Torres Barboza M, Zhang Q, Bochicchio KM, Punch LJ, Bochicchio GV, Ilahi ON (2019). Are patients with perforated peptic ulcers who are negative for *Helicobacter pylori* at a greater risk?. Surg Infect (Larchmt).

[115] Laine L, Hopkins RJ, Girardi LS (1998). Has the impact of *Helicobacter pylori* therapy on ulcer recurrence in the United States been overstated?. Am J Gastroenterol.

[116] Tsimmerman YS (2011). Gastric cancers: a modern approach to the problem. Vestnik chirurgicheskoj gastroenterologii.

[117] Leodolter A, Kulig M, Brasch H, Meyer-Sabellek W, Willich SN, Malfertheiner P (2001). A meta-analysis comparing eradication, healing and relapse rates in patients with *Helicobacter pylori*-associated gastric or duodenal ulcer. Aliment Pharmacol Ther.

[118] Lamb A, Chen LF (2013). Role of the *Helicobacter pylori*-induced inflammatory response in the development of gastric cancer. J Cell Biochem.

[119] Correa P (1992). Human gastric carcinogenesis: a multistep and multifactorial process–first American cancer society award lecture on cancer epidemiology and prevention. Cancer Res.

[120] Rokkas F (2002). *Helicobacter pylori* infection as risk factor of carcinoma of the stomach: current evidence. Russ J Gastroenterol Hepatol Coloproctol.

[121] Yu G, Gail H, Shi J, Klepac-Ceraj V, Paster J, Dye A (2014). Association between upper digestive tract microbiota and cancer-predisposing states in the esophagus and stomach. Cancer Epidemiol Biomarkers Prev.

[122] El Maznya A, Hishmata T, Husseina A, Gaithb D (2019). The prevalence of *Helicobacter pylori* cagA (+ve) among patients with gastric cancer: an Egyptian study. Egypt J Intern Med.

[123] Venerito M, Vasapolli R, Rokkas T, Malfertheiner P (2018). Gastric cancer: epidemiology, prevention, and therapy. Helicobacter.

[124] Merchant SJ, Kim J, Choi AH, Sun V, Chao J, Nelson R (2017). A rising trend in the incidence of advanced gastric cancer in young Hispanic men. Gastric Cancer.

[125] Miftahussurur M, Yamaoka Y, Graham DY (2017). *Helicobacter pylori* as an oncogenic pathogen, revisited. Expert Rev Mol Med.

[126] Ferreira RM, Pereira-Marques J, Pinto-Ribeiro I, Costa JL, Carneiro F, MacHado JC, Figueiredo C (2018). Gastric microbial community profiling reveals a dysbiotic cancer-associated microbiota. Gut.

[127] Camargo MC, Anderson WF, King JB, Correa P, Thomas CC, Rosenberg PS, Eheman CR, Rabkin CS (2011). Divergent trends for gastric cancer incidence by anatomical subsite in US adults. Gut.

[128] Mentis A, Boziki M, Grigoriadis N, Papavassiliou A (2019). *Helicobacter pylori* infection and gastric cancer biology: tempering a double-edged sword. Cel Mol Life Sci.

[129] Ralser A, Dietl A, Jarosch S (2023). *Helicobacter pylori *promotes colorectal carcinogenesis by deregulating intestinal immunity and inducing a mucus-degrading microbiota signature. Gut.

[130] Peng L, Guo Y, Gerhard M, Gao J, Liu Z, Mejías-Luque R (2023). Metabolite alterations and interactions with microbiota in *Helicobacter pylori*-associated gastric lesions. Micobiol Spectr.

[131] Arai J, Niikura R, Hayakawa Y (2021). Use of antibiotics and probiotics reduces the risk of metachronous gastric cancer after endoscopic resection. Biology (Basel).

[132] Niikura R, Hayakawa Y, Nagata N, Miyoshi-Akiayama T, Miyabayashi K, Tsuboi M (2023). Non-*Helicobacter pylori* gastric microbiome modulates prooncogenic responses and is associated with gastric cancer risk. Gastro Hep Adv.

[133] Ford AC, Yuan Y, Forman D, Hunt R, Moayyedi P (2020). *Helicobacter pylori* eradication for the prevention of gastric neoplasia. Cochrane Database Syst Rev.

[134] He C, Peng C, Xu X, Li N, Ouyang Y, Zhu Y (2022). Probiotics mitigate *Helicobacter pylori*-induced gastric inflammation and premalignant lesions in INSGAS mice with the modulation of gastrointestinal microbiota. Helicobacter.

[135] Viazis N, Argyriou K, Kotzampassi K, Christodoulou D, Apostolopoulos P, Georgopoulos S (2022). A four-probiotics regimen combined with a standard *Helicobacter pylori*-eradication treatment reduces side effects and increases eradication rates. Nutrients.

[136] Baryshnikova NV, Ilina AS, Ermolenko EI, Uspenskiy YP, Suvorov AN (2023). Probiotics and autoprobiotics for treatment of *Helicobacter pylori* infection. World J Clin Cases.

[137] Yuan Z, Xiao S, Li S, Suo B, Wang Y, Meng L (2021). The impact of *Helicobacter pylori* infection, eradication therapy, and probiotics intervention on gastric microbiota in young adults. Helicobacter.

[138] Keikha M, Karbalaei M (2021). Probiotics as the live microscopic fighters against *Helicobacter pylori *gastric infections. BMC Gastroenterol.

[139] Zhang L, Zhao M, Fu X (2023). Gastric microbiota dysbiosis and *Helicobacter pylori* infection. Front Microbiol.

[140] Yang C, Liang L, Lv P, Liu L, Wang S, Wang Z, Chen Y (2021). Effects of non-viable Lactobacillus reuteri combining with 14-day standard triple therapy on *Helicobacter pylori* eradication: a randomized double-blind placebo-controlled trial. Helicobacter.

[141] Kamiya S, Yonezawa H, Osaki T (2019). Role of probiotics in eradication therapy for *Helicobacter pylori* infection. Adv Exp Med Biol.

[142] Quin C, Estaki M, Vollman DM, Barnett JA, Gill SK, Gibson DL (2018). Probiotic supplementation and associated infant gut microbiome and health: a cautionary retrospective clinical comparison. Sci Rep.

[143] Suez J, Zmora N, Zilberman-Schapira G, Mor U, Dori-Bachash M, Bashiardes S, Zur M, Regev- Lehavi D, Ben-Zeev Brik R, Federici S (2018). Post-antibiotic gut mucosal microbiome reconstitution is impaired by probiotics and improved by autologous FMT. Cell.

[144] Murros KE, Huynh VA, Takala TM, Saris PEJ (2021). Desulfovibrio bacteria are associated with Parkinson’s disease. Front Cell Infect Microbiol.

[145] Guillemard E, Poirel M, Schäfer F, Quinquis L, Rossoni C, Keicher C, Wagner F, Szajewska H, Barbut F, Derrien M (2021). A randomized, controlled trial: effect of a multi-strain fermented milk on the gut microbiota recovery after *Helicobacter pylori* therapy. Nutrients.

[146] Nabavi-Rad A, Sadeghi A, Yadegar A, Smith S, Zali M (2022). The double-edged sword of probiotic supplementation on gut microbiota structure in *Helicobacter pylori* management. Gut Microbes.

[147] Ye Z, Xia H, Zhang R, Li L, Wu L, Liu X (2020). The efficacy of washed microbiota transplantation on *Helicobacter pylori* eradication: a pilot study. Gastroenterol Res Pract.

[148] Grosen A, Mikkelsen S, Baunwall S, Dahlerup J, Erikstrup L, Hvas C (2023). Risk of *Helicobacter pylori* transmission by faecal microbiota transplantation via oral capsules. Clin Microbiol Infect.

[149] Shah SC, Iyer PG, Moss SF (2021). AGA clinical practice update on the management of refractory *Helicobacter pylori* infection: expert review. Gastroenterology.

